# Changes in Electroencephalography and Cardiac Autonomic Function During Craft Activities: Experimental Evidence for the Effectiveness of Occupational Therapy

**DOI:** 10.3389/fnhum.2020.621826

**Published:** 2020-12-11

**Authors:** Keigo Shiraiwa, Sumie Yamada, Yurika Nishida, Motomi Toichi

**Affiliations:** Department of Human Health Science, Graduate School of Medicine, Kyoto University, Kyoto, Japan

**Keywords:** occupational therapy, frontal midline theta rhythm, autonomic nervous system responses, cardiac sympathetic index, cardiac vagal index, craft activities

## Abstract

Occupational therapy often uses craft activities as therapeutic tools, but their therapeutic effectiveness has not yet been adequately demonstrated. The aim of this study was to examine changes in frontal midline theta rhythm (Fmθ) and autonomic nervous responses during craft activities, and to explore the physiological mechanisms underlying the therapeutic effectiveness of occupational therapy. To achieve this, we employed a simple craft activity as a task to induce Fmθ and performed simultaneous EEG and ECG recordings. For participants in which Fmθ activities were provoked, parasympathetic and sympathetic activities were evaluated during the appearance of Fmθ and rest periods using the Lorenz plot analysis. Both parasympathetic and sympathetic indices increased with the appearance of Fmθ compared to during resting periods. This suggests that a relaxed-concentration state is achieved by concentrating on craft activities. Furthermore, the appearance of Fmθ positively correlated with parasympathetic activity, and theta band activity in the frontal area were associated with sympathetic activity. This suggests that there is a close relationship between cardiac autonomic function and Fmθ activity.

## Introduction

The central role of occupational therapy (OT) is to enhance health and well-being. The “occupation” term in occupational therapy refers to the everyday activities people do to occupy their time and bring meaning and purpose to their lives as individuals, families, and communities [World Federation of Occupational Therapists (WFOT), 2010]. Craft activities have been used as a means of intervention in occupational therapy since the beginning of the profession (Kleinman and Stalcup, 1991; Harris, 2008), especially by occupational therapists working with patients in psychiatric health care (Craik et al., 1998; Griffiths and Corr, 2007). However, previous research on the therapeutic effects of craft activities have primarily been qualitative.

Perruzza and Kinsella's literature review (2010) suggests that creative activities aid in perceptual control, construction of a sense of self, representation, illness experience transformation, acquisition of a sense of purpose, and building social support. Additionally, Leckey (2011) reported that creative activities can have healing and protective effects on mental well-being, which was confirmed by Preminger (2012).

The use of craft activities in occupational therapy has been shown to have some therapeutic effectiveness. Eklund (1999) reported the effectiveness of creative activities in occupational therapy. The OT intervention group had greater improvements in psychological and occupational functioning and global mental health compared to the control group. The randomized controlled trial (Buchain et al., 2003) explored the effects of OT combined with psychopharmacological treatment for clients with schizophrenia. The results showed that patients who received OT along with clozapine had greater improvements in work performance and interpersonal relationships than those who received clozapine alone. Foruzandeh and Parvin (2013) reported a significant improvement in positive and negative symptoms in patients with schizophrenia in the OT group compared to the control group. The results of these previous studies have proven that occupational therapy interventions using craft activities can reduce a variety of psychiatric symptoms and improve occupational functioning. However, there are several phenomena that cannot be studied in the experimental brain research arena due to the need to adapt strictly prescribed methods (Seitamaa-Hakkarainen et al., 2016), and there are few prior studies that provide neuroscientific evidence of therapeutic effects.

The effects of activity-based interventions are thought to originate from the subject's focus on the activity, which can be evaluated using the frontal midline theta rhythm (Fmθ) of an EEG. Fmθ is a 5–7 Hz theta wave that appears in the medial frontal region during extensive cognitive tasks requiring mental concentration (Ishihara and Yoshii, 1972; Ishii et al., 1999). For example, Fmθ reinforcement has reported in meditative states (Aftanas and Golocheikine, 2001), in the pre-fire phase of rifle shooting (Doppelmayr et al., 2008), and when completing implicit tasks (Ishii et al., 2014). During the appearance of Fmθ, more attention is allocated to work tasks and less to monitoring the environment, the self, and the passage of time, making it difficult to interrupt focus on work.

Fmθ is thought to originate in the anterior cingulate cortex (ACC), which is involved in regulation of attention behaviors such as spontaneous attentional functions and conflict resolution (Asada et al., 1999; Ishii et al., 1999, 2014). The ACC also contributes to cognitive control and decision making (Bush, 2009; Mars et al., 2011), and is thought to be responsible for learning the value of a task, selecting tasks based on the learned values, and motivating task execution (Holroyd and Yeung, 2012). Critchley et al. (2004) found that the ACC is involved in regulation of the autonomic nervous system (ANS), with patients containing ACC lesions exhibiting impaired autonomic responses (Critchley et al., 2003). According to studies of brain networks, the autonomic nervous system is regulated by the central autonomic network (CAN) (Verberne and Owens, 1998; Saper, 2002), which includes the ventral medial prefrontal cortex, the ACC, and the insula (Critchley et al., 2011). Representative brain networks include the default mode network (DMN) of the resting state, the executive network (EN) of the task executing state, and the salience network (SN), which examines internal and external information and is involved in switching between the DMN and EN (Damoiseaux et al., 2006; De Luca et al., 2006; Bressler and Menon, 2010; Deco and Corbetta, 2011; Doucet et al., 2011; Menon, 2011). The relationship between brain networks and autonomic activity has also been studied. Beissner et al. (2013) reported that sympathetic-related regions predominate in the EN and SN, while parasympathetic regions predominate in the DMN. Based on these findings, it can be hypothesized that task-related frontal theta rhythms, which reflect the activity of the attentional network (including the ACC), may relate to peripheral autonomic activities.

Frequency-domain analysis (spectral analysis) and time-domain analysis of electrocardiograms (ECG) are often used to evaluate ANS activity during task execution. However, it is difficult to assess sympathetic and parasympathetic nerves separately using frequency-domain analysis (Sawada, 1999; Lahiri et al., 2008: Dodo and Hashimoto, 2015, 2017), while Lorenz plot analysis, a type of time-domain analysis, can measure parasympathetic and sympathetic nervous system activity separately (Toichi et al., 1997). In Lorenz plot analysis, the cardiac sympathetic index (CSI) is used as a measure of sympathetic nervous system activity and the cardiac vagal index (CVI) is used as a measure of parasympathetic nervous system activity. Allen et al. (2007) used Lorenz plot analysis to study performance of a mental arithmetic task requiring active concentration, revealing that execution of this task increased CSI and did not change CVI compared to baseline conditions. In addition, during meditation, both CSI and CVI have been reported to significantly increase during the appearance of Fmθ compared to in the resting state (Kubota et al., 2001). Many studies on Fmθ have used mental tasks, such as a rote computation tasks, so it is not clear how autonomic activity changes during Fmθ-emergent craft activities. We hypothesized that a state of relaxation similar to that of meditation could be achieved in craft activities if a state of concentration of attention was present. Therefore, our study aimed to use Lorenz plot analysis to examine the effect of Fmθ-emergent craft activities on the ANS and evaluate the impact of our results on the potential for therapeutic effects from occupational therapy.

## Materials and Methods

### Participants

Twenty-eight healthy volunteers participated in this study. No participants had cardiac, respiratory, and other diseases that would cause ANS dysfunction. Informed consent was obtained from all participants prior to the experiment. Patients were asked to refrain from eating and drinking (other than water) for 2 h before the experiment. Four participants were excluded based on the following criteria: one for EEG artifacts, one for ECG artifacts, and two for arrhythmias. Ultimately, 24 participants (10 males and 14 females; age range: 20–27 years; mean age: 23.2 ± 1.9 years) were included in the analysis.

### Procedures

#### Task

The task chosen was a form of canvas craft. The task was to thread a thin piece of a single color of cotton yarn through a soft polyethylene mesh (a 35 mm × 80 mm square containing 3 mm × 3 mm holes) using a special needle for metallic yarn in order to create a bookmark. Canvas crafts are widely used in Japan as they are easier than knitting. Before each experiment, we presented samples of canvas handicrafts and practiced making them while explaining the procedure. The experiment was then conducted after participants fully understood the preparation procedure and confirmed that there were no unclear steps.

#### Experiment

Participants experienced a 3-min resting condition (staring at an image of a solid cross), followed by a 7-min craft task (canvas craft), which was repeated for two trials. We selected one condition in which Fmθ was observed during the craft task and defined it as the “Fmθ condition.”

#### EEG Recording and Data Acquisition

**Figure 1:**
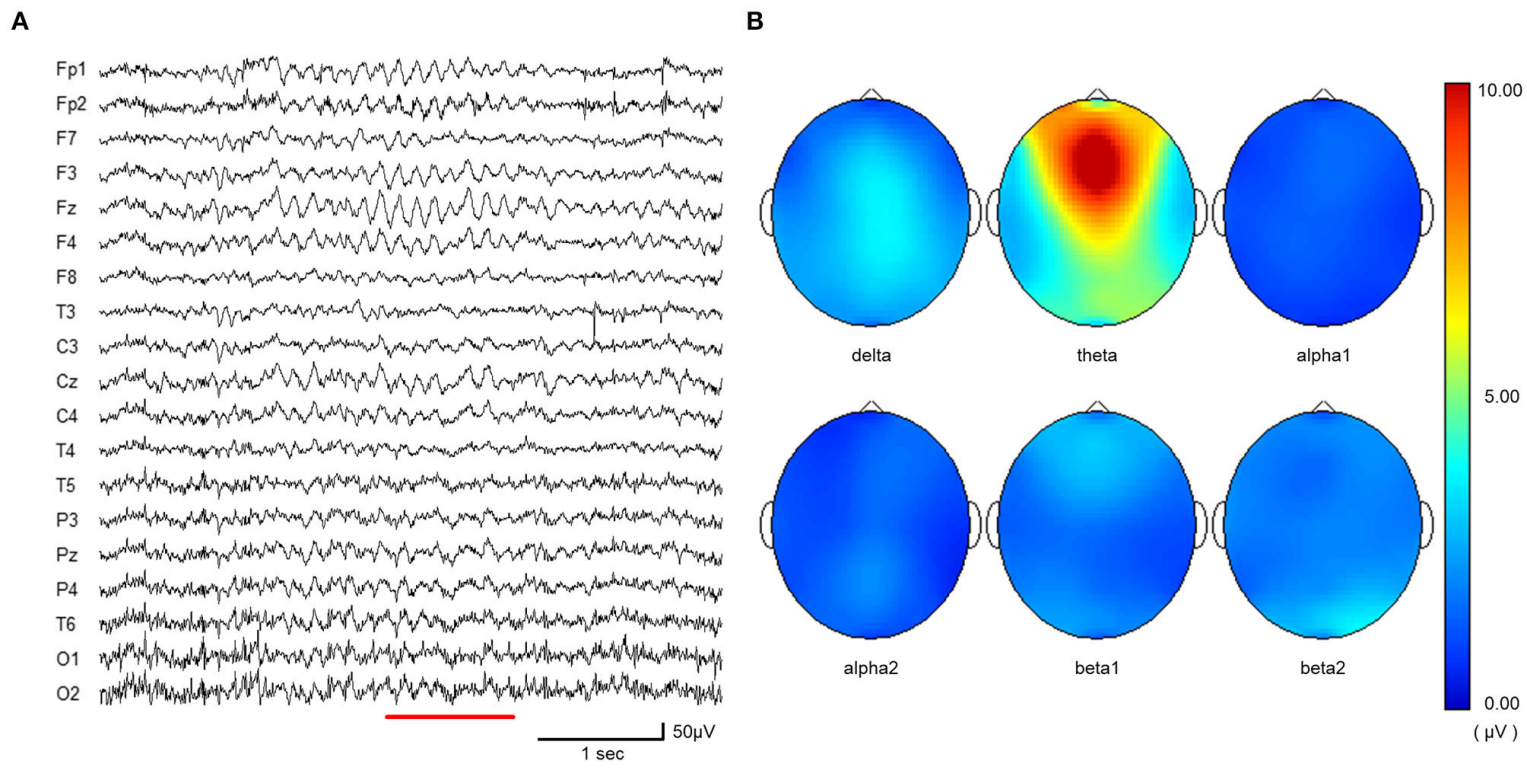
**(A)** EEG sample taken from craft task period showing typical pattern of Fmθ. **(B)** EEG topographic map (from **A**) showing typical peak in theta band in Fz electrode. The spectral density of delta (2.0–4.0 Hz), theta (4.0–8.0 Hz) alpha1 (8.0–10.0 Hz), alpha2 (10.0–13.0 Hz), beta1 (13.0–20.0 Hz), and beta2 (20.0–30.0 Hz) waves for the period of 1.02 s were calculated in amplitude (micro V) using fast Fourier transform (FFT).

BIO-NVX36 (East Medic Co., Ltd., ISHIKAWA, JAPAN) was used for EEG and ECG recordings. EEG recording was done with 19 electrodes using the International 10–20 System and a sampling frequency of 1000 Hz. Electrode resistance was kept below 5 kΩ. Digitized EEG (sampling rate 1000 Hz, bandpass 1.5–100 Hz) was sampled at an epoch of 1.02 s. The criteria of Fmθ were; a train of rhythmic waves, observed at a frequency of 5–7 Hz, having a focal distribution with maximum around the frontal midline in the EEG (Ishihara and Yoshii, 1972; Inouye et al., 1994; Kubota et al., 2001). In this study, theta waves lasting more than 1 s were also selected. ATAMAP II (Kissei Comtec Co., Ltd., Matsumoto, Japan) was used for EEG mapping, and the appearance of Fmθ confirmed by inspecting and mapping the waveforms. The appearance of theta rhythm in the Fz electrode was quantitatively evaluated using spectral analysis software. For spectral analysis, the Fmθ power values were calculated using sampling of 1.02 s epochs, applying a Hanning window to each 1,024-point segment, and using a fast Fourier transform (FFT) to obtain the spectral density per 1.02 s epoch in units of amplitude (μV). Ten of the 24 participants exhibited Fmθ while performing the task. The 14 participants for whom Fmθ did not appear were excluded. In addition, one participant with Fmθ in both the resting and task conditions was ultimately excluded and data from nine participants (three males and six females; age range: 20–25 years; mean age: 22.4 ± 1.6 years) was analyzed. If Fmθ appeared in both trials, the trial in which Fmθ appeared more frequently was selected. An example of EEG and topographical map at the appearance of Fmθ are shown (Figures 1A,B).

#### Autonomic Nervous Response

The ECG signal (Lead 1) was fed into a microcomputer and the inter-beat interval (IBI) triggered by the R-wave measured at a sampling rate of 1 kHz. For the resting condition, a 3-min continuous IBI was used to assess autonomic function. For the Fmθ condition, a 3-min continuous IBI corresponding to the period of Fmθ appearance was selected for the assessment of autonomic function. Lorenz plot analysis was performed using a MaP1060 (NIHONSANTEKU Co., Ltd., Osaka, Japan) to evaluate HRV. The variability of R-R intervals (RRIs) was observed and transformed into an elliptic distribution using Lorenz plots (Toichi et al., 1997) then the length of the longitudinal (L) and transverse (T) axes within the ellipsoid distribution calculated. The cardiac vagal index (CVI) was calculated as a log10 (L × T) transformation and the cardiac sympathetic index (CSI) was calculated as L/T (Toichi et al., 1997).

### Statistical Analyses

The data were analyzed using IBM SPSS version 26. To compare CSI, CVI, and mean RRI values between rest conditions and Fmθ conditions, paired *t*-tests were performed. Cohen's *d* was calculated to determine effect size. In addition, correlation analyses of the number of Fmθ occurrences and power values for CSI, CVI, and changes in CSI and CVI for each period were performed using Pearson's correlation coefficient test.

## Results

### Change of Cardiac Autonomic Activities

**Figure 2:**
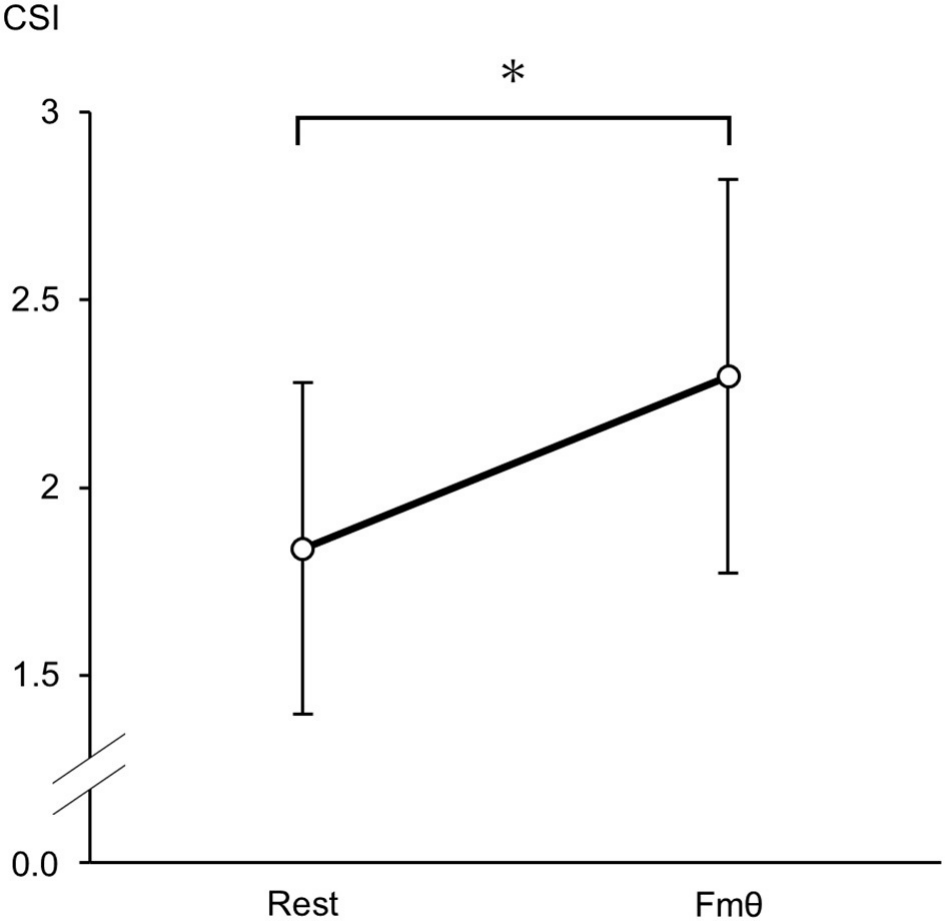
Cardiac sympathetic index (CSI) changes during the rest condition and Fmθ condition. Values are expressed as means and SDs. *p < 0.05.

**Figure 3:**
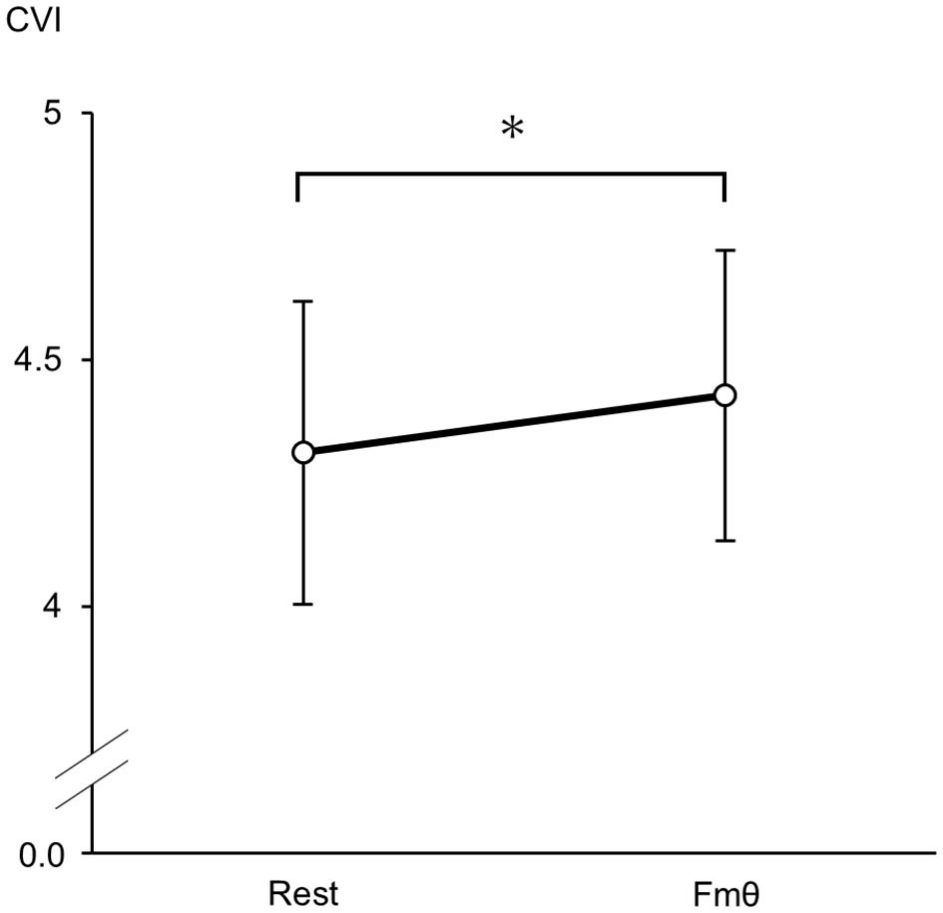
Cardiac vagal index (CVI) changes during the rest condition and Fmθ condition. Values are expressed as means and SDs. *p < 0.05.

Both the cardiac sympathetic index (CSI) and cardiac vagal index (CVI) significantly increased when Fmθ was present compared to rest conditions [CSI: *t*(8) = 2.578, *p* = 0.049, *d* = 0.95; CVI: *t*(8) = 2.323, *p* = 0.033, *d* = 0.39, paired *t*-test]. CSI values during Fmθ conditions (M = 2.30 ± 0.52) were significantly higher than during rest conditions (M = 1.84 ± 0.44; Figure 2). Similarly, CVI values during the Fmθ condition (M = 4.43 ± 0.29) were significantly higher than in the rest condition (M = 4.31, SD = 0.31; Figure 3). In contrast, mean RRI was not significantly different in the Fmθ conditions (M = 877.2 ± 118.6) compared to during rest conditions (M = 897.4 ± 90.1) (*t*(8) = 1.215, *p* = 0.259, *d* = 0.19, paired *t*-test).

### The Correlation of Frontal Theta Activity With CSI and CVI

The mean value of theta power in the Fz electrode was 10.89 ± 1.2 μV, and the mean number of Fmθ appearances was 4.7 ± 3.0. Correlation analysis showed that the power value of Fmθ was positively correlated (*r* = 0.782) with changes in CSI (Table 1). The number of Fmθ appearances was positively correlated with resting CVI (*r* = 0.764) and the Fmθ appearance period (*r* = 0.821).

**Table 1:** Correlations between serum Fmθ power, Fmθ number of appearance and cardiac autonomic activities.

	**Fm** **θ** **power (micro V)**		**Fm** **θ** **number of appearance**	
	***r***	***p*-value**	***r***	***p*-value**
CSI of rest condition	−0.624	0.073	0.041	0.917
CSI of Fmθ condition	0.361	0.339	−0.140	0.719
Change of CSI	0.782	0.013^*^	−0.154	0.693
CVI of rest condition	−0.216	0.576	0.764	0.016^*^
CVI of Fmθ condition	−0.279	0.468	0.821	0.007^*^
Change of CVI	−0.116	0.767	0.055	0.889

*Pearson's correlation coefficient test*,

* *p < 0.05*.

## Discussion

In this study, participants whose Fmθ states appeared during crafting had increased activity of both the sympathetic nervous system, as measured CSI, and the parasympathetic nervous system, as measured by CVI, during Fmθ appearances compared to resting periods. Mental arithmetic tasks have been reported to increase CSI values (Allen et al., 2007; Dodo and Hashimoto, 2019), potentially due to sympathetic activation reflecting mental stress (Lucini et al., 1997). Although an increase in CSI has been associated with a decrease in mean RRI (Pagani et al., 1991), in this study there was no change in mean RRI. This result indicates that a state of relaxation is achieved during craft task completion that is comparable to the resting state. These results also suggest that an increase in CVI may have buffered the impact of the craft activity on CSI values, resulting in lower changes to heart rate. This indicates that crafting activities involve both active, arousal-promoting processes and relaxation processes.

Studies on the effects of meditation and mindfulness have also reported increases in both sympathetic and parasympathetic levels (Jevning et al., 1992; Ditto et al., 2006), suggesting that concentration on crafting tasks can create a similar state. Furthermore, Kubota et al. (2001) reported an increase in both CSI and CVI autonomic activity during the appearance of Fmθ during meditation tasks, which was attributed to a combined concentration-relaxation state. Our study suggests that a similar relaxed-concentration state can be achieved by crafting. The ability of crafting to create a state of relaxation has previously been reported (Reynolds, 2000; Collier, 2011; Preminger, 2012), with a systematic review of arts and crafts activities by Martin et al. (2018) suggesting that these activities contribute to stress reduction and relaxation, all of this were confirmed by our study.

We found that the number of Fmθ appearances was positively correlated with the CVI at rest and during Fmθ appearances. These results suggest that sustained concentration on a task is associated with a relaxed state. However, correlations between Fmθ appearances and resting CVI values indicate potential influence test participant personality traits. In support of this connection, previous research has shown that anxiety and personality traits affect the rate of Fmθ appearance (Inanaga, 1998), which may indicate that those who are more likely to exhibit Fmθ have higher parasympathetic activity. In fact, Tang et al. (2009) reported that Fmθ appearance is correlated with parasympathetic activity, further suggesting a close relationship between the two phenomena.

Also, in our study, the power value of Fmθ was positively correlated with the change in CSI. Moreover, the current proposed source of Fmθ is the region extending from the medial aspect of the prefrontal cortex to the ACC (Asada et al., 1999; Ishii et al., 1999, 2014), with the ACC found to regulate sympathetic activity (Critchley et al., 2003). Finally, overall, our study's results support these findings of previous studies.

Most previous studies on Fmθ have used memorization-, meditation-, and computer game-based tasks, with few reports on Fmθ appearance while performing craft activities. Unlike mental tasks, handicraft activities involve many physical tasks due to the use of tools and objects and associated coordination of eye and hand movements. Performing craft activities requires intimately intertwined, multi-purpose cognition and embodied processing (Huotilainen et al., 2018). In addition, attention is required to successfully complete sequences of performance processes, which likely partly underlies Fmθ induction. The uniqueness of occupational therapy is that the activity involved changes the patient's mental state using objects, freeing the patient from language-based aggression. This may be one mechanism that helps produce the therapeutic effectiveness of relaxed-concentration states in occupational therapy.

While our study confirms the therapeutic effectiveness of crafting activities for some patients, the patient number of Fmθ appearances in this study is about half. Some participants may also exhibit Fmθ states while performing other types of craft beyond our weaving activity, and different types of crafts may vary in their likelihood to induce relaxed concentration states. Based on these caveats, occupational therapists need to provide the most appropriate craft for a given patient.

## Limitations

Multiple limitations were present in our study. First, our sample size was small and the age range was limited to 20–27, limiting our ability to generalize our findings. We chose this age range as this was the group in which Fmθ was most likely to appear. Second, the resting task consisted of looking at a solid cross, and while participants were given instructions to relax, this may not reflect their usual resting state. In fact, one participant exhibited Fmθ during this resting task, indicating that this was a task requiring constant attention. While our resting task was chosen to inhibit eye movement and prevent other artifacts, it apparently may not be a resting state for all participants. However, we recognized that this resting task was more restful than when crafting. These are issues to be considered in future research. This study did not determine the source of Fmθ, but previous studies have shown that ACC is the source of Fmθ. These reports are consistent with our hypothesis, given the role of the ACC in both cognitive function and autonomic control. However, these are only speculations, and there is a need to clarify the current source density and connectivity using the exact low-resolution brain electromagnetic tomography (eLORETA) method (Pascual-Marqui et al., 2011).

## Conclusion

During craft activities in which Fmθ appeared, both parasympathetic and sympathetic indices were increased compared to the resting condition. This result suggests that a certain relaxed-concentration state is achieved by concentrating on craft activities. This can be interpreted as indicating that an appropriate level of concentration for task performance will also cause the same degree of physical relaxation as resting. The results of this study confirm that concentrating on craft activities without being self-conscious has a calming effect and creates a relaxed state, providing evidence for the effectiveness of craft-based occupational therapy.

## Data Availability Statement

The raw data supporting the conclusions of this article will be made available by the authors, without undue reservation.

## Ethics Statement

The studies involving human participants were reviewed and approved by the ethics committee of Kyoto University Graduate School of Medicine (approval number: R1639). The patients/participants provided their written informed consent to participate in this study.

## Author Contributions

KS, SY, and YN contributed to the design, implementation of the research, and the analysis of the results. KS wrote the manuscript with support from MT. All authors contributed to the article and approved the submitted version.

## Conflict of Interest

The authors declare that the research was conducted in the absence of any commercial or financial relationships that could be construed as a potential conflict of interest.
